# A study protocol of external validation of eight COVID-19 prognostic models for predicting mortality risk in older populations in a hospital, primary care, and nursing home setting

**DOI:** 10.1186/s41512-023-00144-2

**Published:** 2023-04-04

**Authors:** Anum Zahra, Kim Luijken, Evertine J. Abbink, Jesse M. van den Berg, Marieke T. Blom, Petra Elders, Jan Festen, Jacobijn Gussekloo, Karlijn J. Joling, René Melis, Simon Mooijaart, Jeannette B. Peters, Harmke A. Polinder-Bos, Bas F. M. van Raaij, Annemieke Smorenberg, Hannah M. la Roi-Teeuw, Karel G. M. Moons, Maarten van Smeden, on behalf of the COOP Consortium, Wilco P. Achterberg, Sifra H. van de Beek, Marian Beekman, Ludo F. M. Beenen, Bram van den Borst, Sebastiaan J. H. Bredie, Frederiek van den Bos, Virgil A. S. H. Dalm, Yvonne M. Drewes, Carline J. van den Dries, Petra J. M. Elders, Miriam C. Faes, Geert-Jan Geersing, Miriam L. Haaksma, Vanessa C. Harris, Ron M. C. Herings, Cees M. P. M. Hertogh, Jacobien J. Hoogerwerf, Jeannette Jacobs-Peters, Steffy Jansen, Anneke G.Julien, Veerle M. G. T. H. van der Klei, Anna Kuranova, P. Hugo M. van der Kuy, Carolien M. J. van der Linden, Anouk M. van Loon, Josephine S. van de Maat, Francesco U. S. Mattace Raso, René J. F. Melis, Julia Minnema, Simon P. Mooijaart, Dennis O. Mook-Kanamori, Mihai G. Netea, Geeske Peeters, Harmke A. Polinder-Bos, Roos S. G. Sablerolles, P. Eline Slagboom, Rosalinde A. L. Smits, Lisanne Tap, Lisa S. van Tol, Hanna C. Willems

**Affiliations:** 1 Julius Center for Health Sciences and Primary Care, University Medical Center, Utrecht University, Utrecht, the Netherlands; 2 Department of Internal Medicine, Radboud University Medical Center, Nijmegen, the Netherlands; 3 Department of General Practice, Amsterdam UMC Location Vrije Universiteit Amsterdam, Amsterdam, the Netherlands; 4 Amsterdam Public Health, Health Behaviors & Chronic Diseases, Amsterdam, the Netherlands; 5 PHARMO Institute for Drug Outcomes Research, Utrecht, the Netherlands; 6 Department of General Practice, Amsterdam Public Health Research Institute, Amsterdam UMC, Amsterdam, the Netherlands; 7 KBO-PCOB, Nieuwegein, the Netherlands; 8 Department of Public Health and Primary Care & Department of Internal Medicine, Section of Gerontology and Geriatrics, Leiden University Medical Center, Leiden, the Netherlands; 9 Department of Medicine for Older People, Amsterdam UMC, Location Vrije Universiteit Amsterdam, Amsterdam, the Netherlands; 10 Amsterdam Public Health, Aging & Later Life, Amsterdam, the Netherlands; 11 Department of Geriatric Medicine, Radboud University Medical Center, Nijmegen, the Netherlands; 12 Department of Internal Medicine, Leiden University Medical Center, Leiden, the Netherlands; 13 Department of Pulmonary Diseases, Radboud Institute for Health Sciences, Radboud University Medical Center, Nijmegen, the Netherlands; 14 Department of Internal Medicine, Section of Geriatric Medicine, Erasmus MC, University Medical Center Rotterdam, Rotterdam, the Netherlands; 15 Department of Internal Medicine, Section of Geriatric Medicine, Amsterdam UMC, Amsterdam, the Netherlands

**Keywords:** COVID-19, COVID-19-related mortality, External validation, Older, Prognostic models

## Abstract

**Background:**

The COVID-19 pandemic has a large impact worldwide and is known to particularly affect the older population. This paper outlines the protocol for external validation of prognostic models predicting mortality risk after presentation with COVID-19 in the older population. These prognostic models were originally developed in an adult population and will be validated in an older population (≥ 70 years of age) in three healthcare settings: the hospital setting, the primary care setting, and the nursing home setting.

**Methods:**

Based on a living systematic review of COVID-19 prediction models, we identified eight prognostic models predicting the risk of mortality in adults with a COVID-19 infection (five COVID-19 specific models: GAL-COVID-19 mortality, 4C Mortality Score, NEWS2 + model, Xie model, and Wang clinical model and three pre-existing prognostic scores: APACHE-II, CURB65, SOFA). These eight models will be validated in six different cohorts of the Dutch older population (three hospital cohorts, two primary care cohorts, and a nursing home cohort). All prognostic models will be validated in a hospital setting while the GAL-COVID-19 mortality model will be validated in hospital, primary care, and nursing home settings. The study will include individuals ≥ 70 years of age with a highly suspected or PCR-confirmed COVID-19 infection from March 2020 to December 2020 (and up to December 2021 in a sensitivity analysis). The predictive performance will be evaluated in terms of discrimination, calibration, and decision curves for each of the prognostic models in each cohort individually. For prognostic models with indications of miscalibration, an intercept update will be performed after which predictive performance will be re-evaluated.

**Discussion:**

Insight into the performance of existing prognostic models in one of the most vulnerable populations clarifies the extent to which tailoring of COVID-19 prognostic models is needed when models are applied to the older population. Such insight will be important for possible future waves of the COVID-19 pandemic or future pandemics.

**Supplementary Information:**

The online version contains supplementary material available at 10.1186/s41512-023-00144-2.

## Background

COVID-19 has a large impact worldwide, causing over 6 million deaths [1], various long-term health effects in a large group of individuals, and an increased burden on healthcare providers and medical institutions. Health outcomes of a COVID-19 infection can be severe, particularly in the older population [2]. In the Netherlands, it is estimated that disproportionately high mortality of 88.8% of all COVID-19 deaths occurred in the older population (≥ 70 years of age) even though they make up only 14% of the total population [3]. Similarly, these mortality proportions in older individuals were also relatively high in hospitalized (60%) and nursing home settings (40%) [3].

Hundreds of prognostic models have been developed to quantify (differences in) mortality risk or other outcomes in COVID-19 patients and to identify individuals at greater risk of developing various future health outcomes [4]. COVID-19 prognostic models have been used to facilitate informed shielding decisions by governments [5], identify higher-risk groups requiring ventilatory or critical care support early to enable targeted recruitment for randomized controlled trials [6], and deliver more personalized, risk-based treatments for which effectiveness is known to vary according to disease severity more precisely [7]. However, despite the development of hundreds of prognostic models, only a few models are of high quality and low risks of bias, according to a critical appraisal in a large living systematic review [4]. For those models that were appraised at a low risk of bias, information about their actual performance in external validation studies is scarce. Further, due to the added complexity of health conditions like frailty [8] and multimorbidity [9] in older individuals, we hypothesize that these prognostic models derived for the general adult population will underperform when validated in an older population.

In this protocol, we describe a comprehensive external validation study to evaluate the predictive performance of eight prognostic models in the older population, defined as individuals aged 70 and older, in hospital, primary care, and nursing home settings. The predictive performance of the prognostic models will be evaluated in a different population than they were derived on, being the older population of individuals aged 70 and older compared to the general adult population. One model will be evaluated in all healthcare settings (hospital care, primary care, and nursing home) to assess predictive performance across settings.

## Methods

We have adhered to the TRIPOD guidelines checklist for external validation studies [10] in reporting this study protocol (Supplementary file 4).

### Selection of COVID-19 prognostic models

**Fig. 1:**
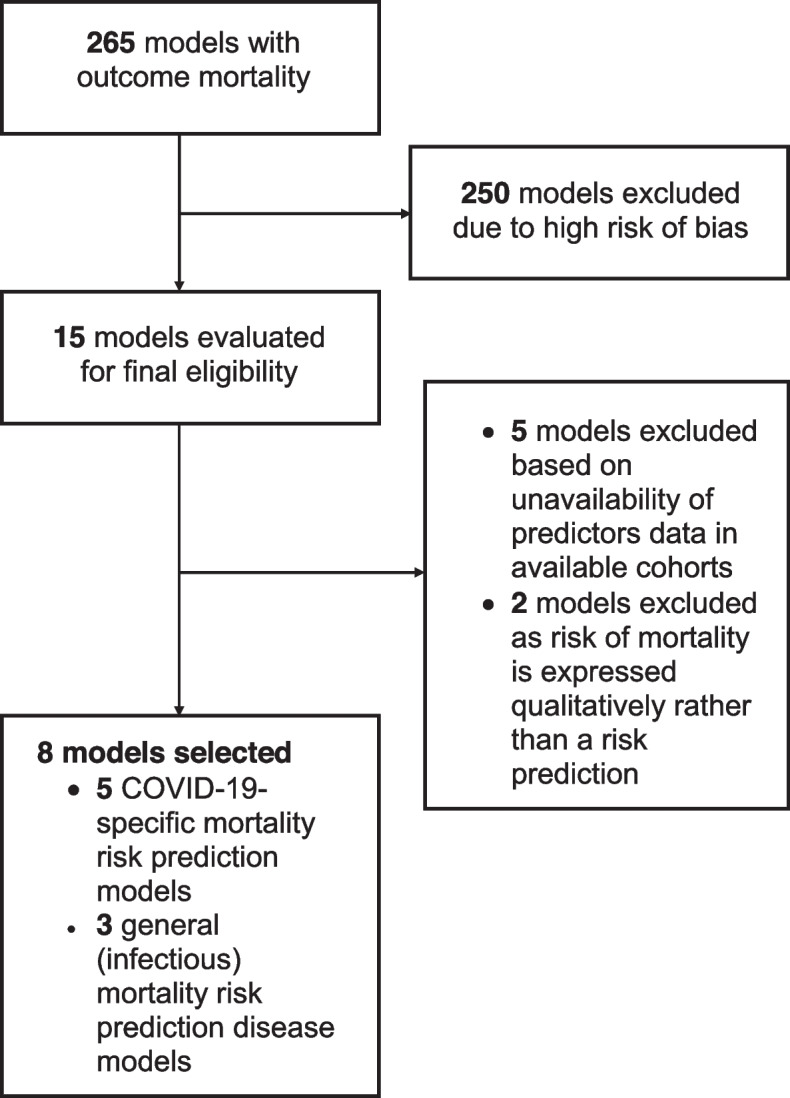
Flowchart for inclusion of prognostic models for external validation

In the living systematic review of diagnostic and prognostic prediction models for COVID-19 (www.covid-precise.org) [4], all published prediction models were reviewed using PROBAST (www.probast.org) [11, 12], a quality or risk of a bias assessment tool for prediction model studies. Using results from the fifth update of this review, we have identified all candidate prognostic models that predict the risk of mortality in individuals with COVID-19 infection with uncertain or low risk of bias. Fifteen candidate models met this criterion of which five prediction models (PRIEST [13], CUCAF-SF [14], CUCA-SF [14], and QCOVID [15] for males and females) were not included for validation due to the unavailability of data on certain predictors in the six cohorts of older patients while two prognostic scores (qSOFA [16] and NEWS [17]) were excluded because they express risk of mortality qualitatively rather than as a risk prediction (Supplementary file 1). Eight prognostic models were included for external validation (Fig. 1). Of these eight models, five were COVID-19-specific (GAL COVID-19 mortality model [18], 4C Mortality Score [19], NEWS2 + model [20], Xie model [21], and Wang clinical model [22]) and were developed in adult COVID-19 populations during the pandemic. Three prognostic models were already existing before COVID-19 pandemic and were used for the prediction of in-hospital mortality risk after admission for any respiratory infections or sepsis (APACHE-II [23], CURB65 [24], and SOFA [25]) (Table 1). The details of eight selected models can be found in Supplementary file 2.

**Table 1:** Overview of selected prognostic models

Model name	Derivation country		Pre-existing or COVID-19 specific	Derivation health care setting	Derivation population	Intended moment of use	Predicted outcome	Predictors		Model type
**GAL-COVID-19-mortality model** [18]	Spain		COVID-19 specific model	Primary care	Adults (≥ 18 years) with confirmed COVID-19 diagnosis	First presentation with COVID-19 infection at general practitioner	Mortality (no prediction horizon reported)	• Age • Sex • Lymphoma/leukemia • Liver disease • Dementia • Ischemic heart disease • Chronic obstructive pulmonary disease • Diabetes mellitus • Chronic kidney disease		Prediction model
**4C-Mortality Score** [19]	UK		COVID-19 specific model	Hospital	Adults (≥ 18 years) with confirmed COVID-19 diagnosis	At hospital admission for COVID-19 infection	In-hospital mortality	• Age • Sex • Respiratory rate • Peripheral oxygen saturation on room air • Glasgow Coma Scale • Urea • C-reactive protein • Number of comorbidities (counted as chronic cardiac disease, chronic respiratory disease (excluding asthma), chronic renal disease, liver disease, dementia, chronic neurological conditions, connective tissue disease, diabetes mellitus, HIV or AIDS, malignancy, obesity)		Points-based score
**NEWS2 + model** [20]		UK	Pre-existing risk stratification score updated for COVID-19 patients	Hospital	Adults (≥ 18 years) admitted to the hospital with a confirmed COVID -19 diagnosis	• At hospital admission for non-nosocomial patients (i.e., community-acquired COVID infection) • At the date of symptom onset for nosocomial patients. If the date of onset was unavailable the date of positive SARS-CoV-2 RT-PCR minus 4 days was used instead	ICU admission or death within 14 days of admission	• Age • Peripheral oxygen saturation • Heart rate • Systolic blood pressure • Body temperature • Alertness • Supplemental oxygen flow rate • Urea • C-reactive protein • Estimated glomerular filtration rate • Neutrophil count • Neutrophil/lymphocyte ratio		Prediction model
**Xie model** [21]		China	COVID-19 specific model	Hospital	Adults (≥ 18 years) admitted to the hospital with a confirmed COVID -19 diagnosis	At hospital admission for COVID-19 infection	In-hospital mortality	• Age • Lactate dehydrogenase • Lymphocyte count • Oxygen saturation		Prediction model
**Wang clinical model** [22]		China	COVID-19 specific model	Hospital	Adults (≥ 18 years) admitted to the hospital with a confirmed COVID-19 diagnosis. Pregnant women were excluded	At hospital admission for COVID-19 infection	In-hospital mortality	• Age • History of hypertension • History of heart disease		Prediction model
**APACHE-II Score** [23]		USA	Pre-existing risk stratification score	Hospital	All newly admitted ICU patients	At admission to ICU	Mortality	• Age • Body temperature • Heart rate • Respiratory rate • Mean arterial pressure • Arterial oxygen tension • pH • Potassium • Sodium • Creatinine • Hematocrit • Leucocyte count • Glasgow Coma Scale • Chronic health evaluation		Points-based score
**CURB-65** [24]		UK, New Zealand, The Netherlands	Pre-existing risk stratification score	Hospital	Patients with community-acquired pneumonia	For triage at the emergency department	Mortality (30 days)		• Age • Alertness (new confusion) • Urea • Respiratory rate • Systolic blood pressure • Diastolic blood pressure	Points-based score
**SOFA Score** [25]		Unclear	Pre-existing risk stratification score	Hospital	All ICU patients	At admission to and during admission at ICU	ICU-mortality		• Arterial oxygen tension • Fraction of inspired oxygen ratio • Glasgow Coma Scale • Mean arterial pressure • Administration of vasopressors • Creatinine • Bilirubin • Platelet count	Points-based score

*COVID-19* Coronavirus disease of 2019, *ICU* Intensive care unit; *RT-PCR* Reverse transcription polymerase chain reaction, *SARS-CoV-2* Severe acute respiratory syndrome coronavirus

### Validation cohorts

Data for this external validation study is collected from six cohorts with older individuals presenting with COVID-19 infection in the Netherlands from three settings: a hospital setting (3 cohorts), primary care setting (2 cohorts), and nursing home setting (1 cohort) (Table 2).

**Table 2:** Details of study cohorts

Healthcare settings	Name of cohort	Description	Models that are externally validated in the cohort
**Hospital setting**	CliniCo	6 Hospitals in the region of Nijmegen in the Netherlands	• GAL-COVID-19 mortality model • 4C Mortality Score • NEWS2 + model • Xie model • Wang clinical model • CURB-65 Score • SOFA Score
	COVID-OLD	15 Hospitals throughout the Netherlands	• GAL-COVID-19 mortality model • 4C Mortality Score • NEWS2 + model • Xie model • Wang clinical model • APACHE-II Score • CURB-65 Score • SOFA Score
	COVID-PREDICT	10 Hospitals throughout the Netherlands	• GAL-COVID-19 mortality model • 4C Mortality Score • NEWS2 + model • Xie model • Wang clinical model • CURB-65 Score • SOFA Score
**Primary care**	JHN/ANH/AHA	General practitioner centers in Utrecht and Amsterdam municipalities in the Netherlands	• GAL-COVID-19 mortality model
	PHARMO	General practitioner centers throughout the Netherlands	• GAL-COVID-19 mortality model
**Nursing homes**	Ysis	Nursing homes throughout the Netherlands	• GAL-COVID-19 mortality model

### Participants

The study participants consist of older individuals (≥ 70 years of age) presenting with highly suspected or reverse transcription polymerase chain reaction (RT-PCR) confirmed COVID-19 from March 2020 to December 2020 in hospital, primary care, and nursing home settings.

Before the widespread use of the RT-PCR test for COVID-19 diagnosis, participants were included using proxy criteria. In the hospital cohorts (CliniCo, COVID-OLD, COVID-PREDICT), the reported respiratory diseases that had COVID-like symptomology are used as an inclusion criterion until 31 March 2020. From April 2020 onwards, a confirmed RT-PCR test for COVID-19 is used as an inclusion criterion. In the primary care cohorts (PHARMO and JHN/ANH/AHA), the participants are included based on free text information and reports of respiratory infections. From June 2020 onwards, ICPC R83.03 for COVID-19 infection is used as an inclusion criterion. The nursing home cohort (Ysis) used RT-PCR as an inclusion criterion. Only participant data on the first presentation or admission of COVID-19 will be included. In the three hospital cohorts, admissions with a duration fewer than 7 days between discharge and readmission will be considered a single hospital admission.

### Outcome

All prediction models have mortality as the predicted outcome (Table 1). In all three hospital cohorts, the outcome is defined as in-hospital mortality. In the primary care and nursing home cohorts, the outcome is defined as 28-day mortality.

### Predictors

Definitions and timing of the predictor variables for the eight models were extracted from original publications (Table 1). Recorded variables in the cohorts are matched, as closely as possible, to the original predictor measurement procedures (Supplementary file 3).

### Statistical analysis

We will externally validate the eight COVID-19 prognostic models in the six cohorts of older patients with COVID-19, aiming to assess their predictive performance when transported from a general adult population to a specific older population. The performance of the GAL-COVID-19 model is assessed across the three healthcare settings. The GAL-COVID-19 mortality model was developed in a primary care setting (general practitioners) and will be validated across different settings (in hospitals, primary care, and nursing homes) [26]. The 4C Mortality Score, NEWS2 + model, Xie model, Wang clinical model, APACHE-II score, CURB-65 score, and SOFA score were developed in hospitalized populations and will be externally validated in the same setting. Evaluation and assessment of the predictive performance of the COVID-19 prognostic models will be performed in each cohort separately. The statistical analysis will be performed in R (version 4.0.0 or later) [27].

#### Descriptive analysis

Participant characteristics and predictor information will be described in all study cohorts (overall and stratified on mortality outcome status) to identify differences in case-mix between the development and validation study populations [28]. These comparisons give insight into the expected model performance and transportability [26].

#### Missing data

The missing data will be described to determine possible reasons for and patterns in missingness [29]. Based on these findings, decisions about the handling of the missing values in the statistical analysis will be made. We anticipate that missing data will be handled using multiple imputations by chained equations using the Full Conditional Specification or Joint Modelling (JOMO) [30]. All variables and outcomes in the final prognostic models are included in the imputation model to ensure compatibility. A total of 50 imputed datasets will then be generated as cohorts are expected to have less than 50% missing values for all relevant variables [31].

#### Assessment of predictive performance

For each prognostic model, we will apply the model according to the authors’ original descriptions and evaluate its predictive performance. We will evaluate discrimination (the model’s ability to distinguish individuals who died after presentation with COVID-19 diagnosis from those who did not) and calibration (the agreement between predicted and observed mortality risks) in each cohort [32]. Discrimination will be assessed in all models by quantifying the area under the receiver operating characteristic curve, i.e., c-statistic [33], and pooled by taking the median c-statistic over imputed datasets and computing dispersion using the interquartile range [34].

Calibration will be assessed by visualizing the calibration of expected versus observed risk using LOESS-smoothed plots on stacked imputed data sets [35]. The GAL-COVID-19 mortality model, NEWS2 + model, Xie model, and Wang clinical model are model equations. For these models, calibration will be assessed in terms of the calibration-in-the-large coefficient and calibration slope [35]. The coefficients are again pooled on a log scale using Rubin’s rules in case of multiple imputed datasets [36]. For each performance measure, for each evaluated model, we will compute the point estimate, standard error, and 95% confidence interval.

#### Decision curve analysis

Decision curve analyses will be performed to quantify the net benefit achieved by each model for predicting the originally intended endpoint across a range of risk thresholds ranging from zero to one [37].

#### Updating

Prediction models showing miscalibration will be adjusted using an intercept update, and predictive performance will be re-assessed for the recalibrated model.

#### Sensitivity analysis

Two sensitivity analyses will be performed to assess the variation in the predictive performance of the eight COVID-19 prognostic models when implemented in different time periods: January 2021 to December 2021 and March 2020 to December 2021. Additionally, predictive performance in estimating the 90-day mortality risk will be evaluated in cohorts that have data available on this outcome (CliniCo, COVID-PREDICT, PHARMO, JHN/ANH/AHA, Ysis).

#### Sample size

Using national statistics from July 2020 to December 2021, the COVID-related mortality fraction for the older population (≥ 70 years) living at home in the Netherlands (including self-reported COVID-19-positive patients) was around 3% [3]. Although we expect higher mortality in nursing homes, hospitals, and ICU admissions, we will assume that an outcome incidence of 3% is the lowest event fraction that we will encounter for the current prediction model validation study. We take this fraction as a starting point for the sample size calculations, which are based on sample size calculation recommendations for external validation studies by Riley and colleagues [38]. Since we are considering various models, we base our sample size calculation around a general scenario that is reasonably applicable to all cases. Based on the distributions of predicted risk of mortality that were previously reported for some of the cohorts [8, 39], we assume the distribution of the linear predictor to be approximately N (− 3.9,1) [38]. Based on the calculations, a sample size of 754 will be required per cohort to validate an assumably well-calibrated model with a calibration-in-the-large coefficient of 0 and calibration slope of 1, and an assumed target standard error of calibration slope of 0.02. The target standard error of the calibration slope was chosen to ensure the evaluation of calibration with a higher precision than the typically used value of 0.05.

## Discussion

This external validation study will assess the predictive performance of pre-existing clinical and COVID-19-specific prognostic models in the older population. Our study will reveal the validity of these COVID-19 prognostic models in one of the most vulnerable populations, the older population, as well as give insight into the requirements for tailored prediction models for the older population in future COVID-19 waves or pandemics.

While previous studies have largely focused on external validation of COVID-19 prediction models in the general adult population [14, 40, 41], this external validation study will focus on evaluating the performance of existing COVID-19 prognostic models specifically in the older population which represents the highest proportion of hospitalized COVID-19 patients and the highest mortality [3].

External validation in multiple cohorts across different healthcare settings allows for the assessment of between-setting heterogeneity in predictive performance and applicability at different points of care in older COVID-19 patients. This is one of the first studies to evaluate prognostic models for COVID-19 disease in individuals living in nursing homes.

### Challenges and limitations

There are certain challenges that we anticipate encountering while conducting this research. Definitions of participant inclusion and predictor measurement procedures are expected to differ across cohorts. Although we have carefully planned to collect information on the definition of a COVID-19 infection and predictor measurements for each cohort, we cannot rule out the possibility of heterogeneity occurring across different settings and its effects on model performance [42]. However, this heterogeneity resembles practical conditions encountered in clinical practice and thus provides relevant knowledge on the anticipated performance of the models in clinical practice.

A limitation of this study is that not all low risk of bias COVID-19 prognostic models identified by the living systematic review could be included in this external validation. This was due to the lack of predictor information available in the cohorts. Similarly, predictor measurements and incidence of mortality due to COVID-19 can vary over time due to newer variants, better medical management, and improved vaccination coverage [43]. These temporal changes can potentially limit the predictive performance of the prognostic models being investigated in the current study.

## Conclusion

External validation of newer and existing COVID-19 prognostic models can provide evidence about their predictive performance when implemented in the older population as tools of effective risk stratification and in aiding decision-making for targeted and timely clinical interventions.

## Supplementary Information

**Additional file 1: Supplementary file 1.** Description of excluded prognostic models.

**Additional file 2: Supplementary file 2.** Prediction models and risk scores.

**Additional file 3: Supplementary file 3.** Predictor measurements in derivation and validation cohorts.

**Additional file 4: Supplementary file 4.** TRIPOD checklist for validation of prediction model

## Data Availability

The datasets generated and/or analyzed during the current study are not publicly available due to confidentiality. COVID-OLD, CliniCo, COVID-PREDICT, PHARMO, and JHN/ANH/AHA are available on reasonable request.

## Abbreviations

PCR: Polymerase chain reaction
APACHE score: Acute Physiology and Chronic Health Evaluation score
NEWS score: National Early Warning Score
SOFA score: Sequential organ failure assessment score
PROBAST: Prediction model Risk of Bias Assessment Tool
RT-PCR: Reverse transcription polymerase chain reaction
JOMO: Joint modeling
LOESS: Locally estimated scatterplot smoothing

## References

[1] WHO. WHO coronavirus (COVID-19) dashboard 2022. Overview. Available from: https://covid19.who.int.

[2] Smorenberg A, Peters EJ, van Daele P, Nossent EJ, Muller M (2021). How does SARS-CoV-2 targets the elderly patients? A review on potential mechanisms increasing disease severity. Eur J Intern Med.

[3] Rijksoverheid. Sterfte 2022. Date accessed: April 2022. Available from: https://coronadashboard.rijksoverheid.nl/landelijk/sterfte.

[4] Wynants L, Van Calster B, Collins GS, Riley RD, Heinze G, Schuit E (2020). Prediction models for diagnosis and prognosis of covid-19: systematic review and critical appraisal. BMJ.

[5] Smith GD, Spiegelhalter D (2020). Shielding from COVID-19 should be stratified by risk. BMJ.

[6] Furlow B (2020). COVACTA trial raises questions about tocilizumab’s benefit in COVID-19. Lancet Rheumatol.

[7] Beigel JH, Tomashek KM, Dodd LE, Mehta AK, Zingman BS, Kalil AC (2020). Remdesivir for the treatment of COVID-19 - final report. N Engl J Med.

[8] Blomaard LC, van der Linden CMJ, van der Bol JM, Jansen SWM, Polinder-Bos HA, Willems HC (2021). Frailty is associated with in-hospital mortality in older hospitalised COVID-19 patients in the Netherlands: the COVID-OLD study. Age Ageing.

[9] Barnett K, Mercer SW, Norbury M, Watt G, Wyke S, Guthrie B (2012). Epidemiology of multimorbidity and implications for health care, research, and medical education: a cross-sectional study. Lancet.

[10] Collins GS, Reitsma JB, Altman DG, Moons KG (2015). Transparent Reporting of a multivariable prediction model for Individual Prognosis or Diagnosis (TRIPOD): the TRIPOD statement. Ann Intern Med..

[11] Moons KGM, Wolff RF, Riley RD, Whiting PF, Westwood M, Collins GS (2019). PROBAST: a tool to assess risk of bias and applicability of prediction model studies: explanation and elaboration. Ann Intern Med.

[12] Wolff RF, Moons KGM, Riley RD, Whiting PF, Westwood M, Collins GS (2019). PROBAST: a tool to assess the risk of bias and applicability of prediction model studies. Ann Intern Med.

[13] Goodacre S, Thomas B, Sutton L, Burnsall M, Lee E, Bradburn M (2021). Derivation and validation of a clinical severity score for acutely ill adults with suspected COVID-19: the PRIEST observational cohort study. PLoS ONE.

[14] Bradley P, Frost F, Tharmaratnam K, Wootton DG, Research NWCOfR (2020). Utility of established prognostic scores in COVID-19 hospital admissions: multicentre prospective evaluation of CURB-65, NEWS2 and qSOFA. BMJ Open Respir Res.

[15] Clift AK, Coupland CAC, Keogh RH, Diaz-Ordaz K, Williamson E, Harrison EM (2020). Living risk prediction algorithm (QCOVID) for risk of hospital admission and mortality from coronavirus 19 in adults: national derivation and validation cohort study. BMJ.

[16] Seymour CW, Liu VX, Iwashyna TJ, Brunkhorst FM, Rea TD, Scherag A (2016). Assessment of Clinical Criteria for Sepsis: for the third international consensus definitions for sepsis and septic shock (sepsis-3). JAMA.

[17] Royal College of Physicians (2012). National Early Warning Score (NEWS): standardising the assessment of acute- illness severity in the NHS. Report of a working party.

[18] Gude-Sampedro F, Fernandez-Merino C, Ferreiro L, Lado-Baleato O, Espasandin-Dominguez J, Hervada X (2021). Development and validation of a prognostic model based on comorbidities to predict COVID-19 severity: a population-based study. Int J Epidemiol.

[19] Knight SR, Ho A, Pius R, Buchan I, Carson G, Drake TM (2020). Risk stratification of patients admitted to hospital with COVID-19 using the ISARIC WHO Clinical Characterisation Protocol: development and validation of the 4C Mortality Score. BMJ.

[20] Carr E, Bendayan R, Bean D, Stammers M, Wang W, Zhang H (2021). Evaluation and improvement of the National Early Warning Score (NEWS2) for COVID-19: a multi-hospital study. BMC Med.

[21] Xie J, Hungerford D, Chen H, Abrams ST, Li S, Wang G (2020). Development and external validation of a prognostic multivariable model on admission for hospitalized patients with COVID-19.

[22] Wang K, Zuo P, Liu Y, Zhang M, Zhao X, Xie S (2020). Clinical and laboratory predictors of in-hospital mortality in patients with coronavirus disease-2019: a cohort study in Wuhan, China. Clin Infect Dis.

[23] Knaus WA, Draper EA, Wagner DP, Zimmerman JE (1985). APACHE II: a severity of disease classification system. Crit Care Med.

[24] Lim WS, van der Eerden MM, Laing R, Boersma WG, Karalus N, Town GI, Lewis SA, Macfarlane JT (2003). Defining community acquired pneumonia severity on presentation to hospital: an international derivation and validation study. Thorax.

[25] Vincent JL, Moreno R, Takala J, Willatts S, De Mendonça A, Bruining H, Reinhart CK, Suter PM, Thijs LG (1996). The SOFA (Sepsis-related Organ Failure Assessment) score to describe organ dysfunction/failure. On behalf of the Working Group on Sepsis-Related Problems of the European Society of Intensive Care Medicine. Intensive Care Med.

[26] Justice AC, Covinsky KE, Berlin JA (1999). Assessing the generalizability of prognostic information. Ann Intern Med.

[27] Team Rc (2022). R: A language and environment for statistical computing.

[28] Debray TP, Vergouwe Y, Koffijberg H, Nieboer D, Steyerberg EW, Moons KG (2015). A new framework to enhance the interpretation of external validation studies of clinical prediction models. J Clin Epidemiol.

[29] Donders AR, van der Heijden GJ, Stijnen T, Moons KG (2006). Review: a gentle introduction to imputation of missing values. J Clin Epidemiol.

[30] James Carpenter MK (2012). Multiple imputation and its application.

[31] White IR, Royston P, Wood AM (2011). Multiple imputation using chained equations: issues and guidance for practice. Stat Med.

[32] Moons KG, Altman DG, Vergouwe Y, Royston P (2009). Prognosis and prognostic research: application and impact of prognostic models in clinical practice. BMJ.

[33] Steyerberg EW, Vickers AJ, Cook NR, Gerds T, Gonen M, Obuchowski N (2010). Assessing the performance of prediction models: a framework for traditional and novel measures. Epidemiology.

[34] Marshall A, Altman DG, Holder RL, Royston P (2009). Combining estimates of interest in prognostic modelling studies after multiple imputation: current practice and guidelines. BMC Med Res Methodol.

[35] Van Calster B, McLernon DJ, van Smeden M, Wynants L, Steyerberg EW, Topic Group 'Evaluating diagnostic t (2019). Calibration: the Achilles heel of predictive analytics. BMC Med.

[36] Rubin DB (1987). Multiple imputation for nonresponse in surveys.

[37] Vickers AJ, van Calster B, Steyerberg EW (2019). A simple, step-by-step guide to interpreting decision curve analysis. Diagn Progn Res.

[38] Riley RD, Debray TPA, Collins GS, Archer L, Ensor J, van Smeden M (2021). Minimum sample size for external validation of a clinical prediction model with a binary outcome. Stat Med.

[39] Rutten JJS, van Loon AM, van Kooten J, van Buul LW, Joling KJ, Smalbrugge M (2020). Clinical suspicion of COVID-19 in nursing home residents: symptoms and mortality risk factors. J Am Med Dir Assoc.

[40] Gupta RK, Marks M, Samuels THA, Luintel A, Rampling T, Chowdhury H (2020). Systematic evaluation and external validation of 22 prognostic models among hospitalised adults with COVID-19: an observational cohort study. Eur Respir J.

[41] de Jong VMT, Rousset RZ, Antonio-Villa NE, Buenen AG, Van Calster B, Bello-Chavolla OY (2022). Clinical prediction models for mortality in patients with covid-19: external validation and individual participant data meta-analysis. BMJ.

[42] Luijken K, Groenwold RHH, Van Calster B, Steyerberg EW, van Smeden M (2019). Impact of predictor measurement heterogeneity across settings on the performance of prediction models: a measurement error perspective. Stat Med.

[43] Watson OJ, Barnsley G, Toor J, Hogan AB, Winskill P, Ghani AC (2022). Global impact of the first year of COVID-19 vaccination: a mathematical modelling study. Lancet Infectious Dis..

