# Supplementary material for: A study protocol of external validation of eight COVID-19 prognostic models for predicting mortality risk in older populations in a hospital, primary care, and nursing home setting
Source: Diagn Progn Res. 2023 Apr 4;7:8. doi: 10.1186/s41512-023-00144-2 (PMC10069944; doi:10.1186/s41512-023-00144-2)
Supplement: Supplementary file 1 — Additional file 1: Supplementary file 1. Description of excluded prognostic models. [file 41512_2023_144_MOESM1_ESM.pdf]

Supplementary file 1: Description of excluded prognostic models

Five models were excluded from this validation study due to the unavailability of certain predictors in the validation cohorts and two models were excluded because they express the risk of mortality qualitatively rather than as a risk prediction. The details of these models and the reason for their exclusion are listed in the table below.

Supplementary Table 1: Characteristics of candidate prognostic models for COVID-19 not included in current external validation study

Model Name	Pre-existing or COVID-Specific	Model Outcome	Reason for exclusion
**Q-COVID (Two models separately for males and females)**	COVID-specific	Mortality	Information on Townsend deprivation index, ethnicity, and domicile not available in validation cohorts.
**PRIEST**	COVID-specific	Mortality	Information on performance status and major organ support not available in most validation cohorts.
**CUCAF-SF**	COVID-specific	Mortality	Information on consolidation on chest radiography not available in most validation cohorts.
**CUCA-SF**	COVID-specific	Mortality	Information on consolidation on chest radiography not available in most validation cohorts.
**NEWS**	Pre-existing risk stratification score	Mortality	Express risk of mortality qualitatively rather than as a risk prediction.
**qSOFA**	Pre-existing risk stratification score	Mortality	Express risk of mortality qualitatively rather than as a risk prediction.
