# Supplementary material for: A study protocol of external validation of eight COVID-19 prognostic models for predicting mortality risk in older populations in a hospital, primary care, and nursing home setting
Source: Diagn Progn Res. 2023 Apr 4;7:8. doi: 10.1186/s41512-023-00144-2 (PMC10069944; doi:10.1186/s41512-023-00144-2)
Supplement: Supplementary file 2 — Additional file 2: Supplementary file 2. Prediction models and risk scores. [file 41512_2023_144_MOESM2_ESM.pdf]

Supplementary file 2: Prediction models and risk scores

The details of the eight prognostic models selected for the external validation study are described in this section. This includes the model equations or score tables, approximated mortality risks, comparison tables between derivation and validation settings, and model development study references.

1) GAL-COVID-19 mortality model

Model equation

Logit(mortality) = -9.522 + 0.095 x age + 0.889 x sex + 1.463 x lymphoma/leukemia + 0.703 x liver disease + 0.495 x ischemic heart disease + 0.564 x dementia + 0.548 x Chronic obstructive pulmonary disease + 0.613 x Diabetes - 0.828 x Chronic kidney disease

Comparison between derivation and validation settings

	**Derivation settings**	**Validation hospital cohorts**		
		**CliniCo**	**COVID-OLD**	**COVID-Predict**
**Country**	Galicia, Spain	The Netherlands	The Netherlands	The Netherlands
**Healthcare setting**	Primary care	Hospital	Hospital	Hospital
**Population**	• Adults (≥18 years)<br>• Confirmed COVID -19 diagnosis	• Older population (≥70 years)<br>• Confirmed COVID -19 diagnosis	• Older population (≥70 years)<br>• Confirmed COVID -19 diagnosis	• Older population (≥70 years)<br>• Confirmed COVID -19 diagnosis
**Predicted outcome and time of horizon**	Mortality (prediction horizon not reported)	In-hospital mortality	In-hospital mortality	In-hospital mortality

	**Derivation settings**	**Validation primary care and nursing home cohorts**		
		**JHN, ANH, and AHA**	**PHARMO**	**YSIS**
**Country**	United Kingdom	The Netherlands	The Netherlands	The Netherlands
**Healthcare setting**	Primary care	Primary care	Primary care	Nursing home
**Population**	• Adults (≥18 years)<br>• Confirmed COVID -19 diagnosis	• Older population (≥70 years)<br>• Confirmed COVID -19 diagnosis	• Older population (≥70 years)<br>• Confirmed COVID -19 diagnosis	• Older population (≥70 years)<br>• Confirmed COVID -19 diagnosis
**Predicted outcome and time of horizon**	Mortality (prediction horizon not reported)	28-day mortality	28-day mortality	28-day mortality

Reference

Francisco Gude-Sampedro , Carmen Fernández-Merino, Lucía Ferreiro, Óscar Lado-Baleato, Jenifer Espasandín-Domínguez, Xurxo Hervada 7 , Carmen M Cadarso 6 , Luis Valdés. Development and validation of a prognostic model based on comorbidities to predict COVID-19 severity: a population-based study. International Journal of Epidemiology. 2021 Mar 3;50(1):64-74. DOI: 10.1093/ije/dyaa209 https://pubmed.ncbi.nlm.nih.gov/33349845/

2) 4C Mortality Score

Score calculation

In-hospital mortality score = 2 x age 50 to 59 + 4 x age 60 to 69 + 6 x age 70 to 79 + 7 x age > 80 + 1 x male + 1 x one comorbidity + 2 x multiple comorbidities + 1 x respiratory rate (breath/minute) 20 to 29 + 2 x respiratory rate (breath/minute) ≥ 30 + 2 x oxygen saturation on room air (%) <92 + 2 x Glasgow coma scale < 15 + urea (mmol/L) 7 to 14 + 3 x urea (mmol/L) >14 + 1 x C-reactive protein (mg/L) 50 to 99 + 2 x C-reactive protein (mg/L) ≥ 100)

Comorbidities assessed: Chronic cardiac disease, chronic respiratory disease (excluding asthma), chronic renal disease (estimated glomerular filtration rate ≤30), liver disease (moderate to severe), dementia, chronic neurological conditions, connective tissue disease, diabetes, acquired immunodeficiency virus/ Human immunodeficiency virus, malignancy, obesity (clinically defined).

Approximated in-hospital mortality risks

4C Mortality Score	Mortality risk (%)
**0**	0.001
**1**	0.3
**2**	0.8
**3**	2.3
**4**	4.8
**5**	7.5
**6**	7.8
**7**	11.7
**8**	14.4
**9**	19.2
**10**	22.9
**11**	26.9
**12**	32.9

**13**	40.1
**14**	44.6
**15**	51.6
**16**	59.1
**17**	66.1
**18**	75.8
**19**	77.4
**20**	82.9
**21**	87.5

Comparison between derivation and validation settings

	**Derivation settings**	**Validation hospital cohorts**		
		**CliniCo**	**COVID-OLD**	**COVID-Predict**
**Country**	United Kingdom	The Netherlands	The Netherlands	The Netherlands
**Healthcare setting**	Hospital	Hospital	Hospital	Hospital
**Population**	• Adults (≥18 years)<br>• Confirmed COVID -19 diagnosis	• Older population (≥70 years)<br>• Confirmed COVID -19 diagnosis	• Older population (≥70 years)<br>• Confirmed COVID -19 diagnosis	• Older population (≥70 years)<br>• Confirmed COVID -19 diagnosis
**Predicted outcome and time of horizon**	In-hospital mortality	In-hospital mortality	In-hospital mortality	In-hospital mortality

Reference

Knight SR, Ho A, Pius R, Buchan I, Carson G, Drake TM, Dunning J, Fairfield CJ, Gamble C, Green CA, Gupta R. Risk of patients admitted to hospital with Covid-19 using the ISARIC WHO Clinical Characterization Protocol: development and validation of the 4C Mortality score. BMJ. 9;370. https://www.bmj.com/content/370/bmj.m3339

3) NEWS2+ model

Model equation

Logit(mortality) = Intercept + 0.145 x C-reactive protein - 0.232035 x estimated glomerular filtration rate + 0.088656 x neutrophils + 0.057681 x urea + 0.066401 x neutrophil-lymphocyte ratio – 0.198729 x oxygen saturation + 0.329529 x supplemental oxygen flow rate + 0.417074 x NEWS2 score + 0.279495 x age

Comparison between derivation and validation settings

Derivation settings		Validation hospital cohorts		
		**CliniCo**	**COVID-OLD**	**COVID-Predict**
**Country**	United Kingdom	The Netherlands	The Netherlands	The Netherlands
**Healthcare setting**	Hospital	Hospital	Hospital	Hospital
**Population**	• Adults (≥18 years)<br>• Confirmed COVID -19 diagnosis	• Older population (≥70 years)<br>• Confirmed COVID -19 diagnosis	• Older population (≥70 years)<br>• Confirmed COVID -19 diagnosis	• Older population (≥70 years)<br>• Confirmed COVID -19 diagnosis
**Predicted outcome and time of horizon**	ICU admission or mortality within 14 days of admission	In-hospital mortality	In-hospital mortality	In-hospital mortality

Reference

Carr, E., Bendayan, R., Bean, D. *et al.* Evaluation and improvement of the National Early Warning Score (NEWS2) for COVID-19: a multi-hospital study. *BMC Med* **19,** 23 (2021). https://doi.org/10.1186/s12916-020-01893-3

4) Wang Clinical model

Model equation

Logit(mortality) = -8.6 + 0.10 x age + 0.60 x history of hypertension + 1.11 x history of CHD

Comparison between derivation and validation settings

	Derivation settings	Validation hospital cohorts		
		**CliniCo**	**COVID-OLD**	**COVID-Predict**
**Country**	China	The Netherlands	The Netherlands	The Netherlands
**Healthcare setting**	Hospital	Hospital	Hospital	Hospital
**Population**	• Adults (≥18 years)<br>• Confirmed COVID -19 diagnosis	• Older population (≥70 years)<br>• Confirmed COVID -19 diagnosis	• Older population (≥70 years)<br>• Confirmed COVID -19 diagnosis	• Older population (≥70 years)<br>• Confirmed COVID -19 diagnosis

Predicted outcome and time of horizon	In-hospital mortality	In-hospital mortality	In-hospital mortality	In-hospital mortality

Reference

Wang K, Zuo P, Liu Y, Zhang M, Zhoa X, Xie S, Zhang H, Chen X, Liu C. Clinical and laboratory predictors of in-hospital mortality in patients with COVID_19: a cohort study in Wuhan, China. 2020 May3. https://pubmed.ncbi.nlm.nih.gov/32361723/

5) Xie model

Model equation

Logit(mortality) = -4.559 + 0.047 x age + 0.003 x Lactate dehydrogenase (U/L) - 1.094 x lymphocyte count (10^9/L) - 0.098 x SPO_2 (%)

Comparison between derivation and validation settings

	Derivation settings	Validation hospital cohorts		
		CliniCo	COVID-OLD	COVID-Predict
**Country**	China	The Netherlands	The Netherlands	The Netherlands
**Healthcare setting**	Hospital	Hospital	Hospital	Hospital
**Population**	• Adults (≥18 years)<br>• Confirmed COVID -19 diagnosis	• Older population (≥70 years)<br>• Confirmed COVID -19 diagnosis	• Older population (≥70 years)<br>• Confirmed COVID -19 diagnosis	• Older population (≥70 years)<br>• Confirmed COVID -19 diagnosis
**Predicted outcome and time of horizon**	In-hospital mortality	In-hospital mortality	In-hospital mortality	In-hospital mortality

Reference

Xie J, Hungerford D, Chen H, Abrams ST, Li S, Wang G, Wang Y, Kang H, Bonnett L, Zheng R, Li X, Tong Z, Du B, Qiu H, Toh C-H. Development and external validation of a prognostic multivariable model on admission for hospitalized patients with COVID-19. medRxiv Cold Spring Harbor Laboratory Press; 2020; :2020.03.28.20045997 https://www.medrxiv.org/content/10.1101/2020.03.28.20045997v2

6) APACHE-II Score

APACHE-II Mortality Score = Age points[1] + Acute Physiology Score[2] + Chronic health[3]

Age points[1]

Age Points	0	+2	+3	+5	+6
Age (years)	≤44	45-54	55-64	65-74	>74

Acute Physiology Score[2]

Acute physiology score is the sum of the 12 individual variable points: Rectal temperature, mean arterial temperature, respiratory rate, arterial blood gases (arterial pH, oxygenation), venous blood variables (hematocrit, white blood cells, potassium, and creatinine), Glasgow coma scale. –

	Physiological Variables		Physiology score points								
			+4	+3	+2	+1	0	+1	+2	+3	+4
1	Rectal temperature		≥41	39 – 40.9	-	38.5 – 38.9	36 – 38.4	34 – 35.9	32 – 33.9	30 – 31.9	≤29.9
2	Mean arterial pressure (mm Hg)		≥160	130 – 159	110 – 129	-	70 – 109	-	50 – 69	-	≤49
3	Heart rate		≥180	140 – 179	110 – 139	-	70 – 109	-	55 – 69	40 – 54	≤39
4	Respiratory rate		≥50	35 – 49	-	25 – 34	12 – 24	10 – 11	6 – 9	-	≤5
5	Oxygenation	Fraction of inspired oxygen ≥0.5	≥500	350 – 499	200 – 349	-	<200	-	-	-	-
		Fraction of inspired oxygen <0.5: use Partial pressure of oxygen	-	-	-	-	>70	61 – 70	-	55 – 60	<55
6	Arterial pH		≥7.7	7.6 – 7.69	-	7.5 – 7.59	7.33 – 3.49	-	7.25 – 7.32	7.15 – 7.24	<7.5
7	Sodium (mMol/L)		≥180	160 – 179	155 – 159	150 – 154	130 – 149	-	120 – 129	111 – 119	≤110
8	Potassium (mMol/L)		≥7	6 – 6.9	-	5.5 – 5.9	3.5 – 5.4	3 – 3.4	2.5 – 2.9	-	<2.5
9	Serum Creatinine (mg/dL)		≥3.5	2 – 3.4	1.5 – 1.9	-	0.6 – 1.4	-	<0.6	-	-
10	Hematocrit (%)		≥60	-	50 – 59.9	46 – 49.9	30 – 45.9	-	20 – 29.9	-	<20
11	White blood count (in 1000s)		≥40	-	20 – 39.9	15 – 19.9	3 – 14.9	-	1 – 2.9	-	<1
12	Glasgow Coma scale (GCS)		Score = 15 minus actual GCS								

Chronic health[3]

Chronic health status points: two points if an elective postoperative patient with immunocompromise or history of severe organ insufficiency: five points for a non-operative patient or emergency post-operative patient with immunocompromise (i.e., due to chemotherapy, radiation, high dose steroid therapy, or advanced leukemia, lymphoma, or AIDS) or severe organ insufficiency (liver cirrhosis, portal hypertension, class IV heart failure, severe respiratory disease, dialysis-dependent)

Comparison between derivation and validation settings

Derivation settings		Validation hospital cohorts
		**COVID-OLD**
**Country**	United States	The Netherlands
**Healthcare setting**	Hospital	Hospital
**Population**	Intensive care admissions	• Older population (≥70 years)<br>• Confirmed COVID -19 diagnosis
**Predicted outcome and time of horizon**	ICU mortality	In-hospital mortality

Approximated in-hospital mortality risks

APACHE II Score	Mortality risk
**0-4**	4%
**5-9**	8%
**10-14**	15%
**15-19**	25%
**20-24**	40%
**25-29**	55%
**30-34**	73%
**>34**	85%

Reference

Knaus WA, Draper EA, Wagner DP, Zimmerman JE. APACHE II: a severity of disease classification system. Crit Care Med. 1985 Oct;13(10):818-29.

7) CURB-65 score

Score calculation

Characteristics	No	Yes
**Confusion**	0	+1
**Blood urea nitrogen >19 mg/dL (>7 mmol/L)**	0	+1

**Respiratory rate ≥ 30**	0	+1
**Systolic BP < 90 mmHg or Diastolic BP ≤ 60 mmHg**	0	+1
**Age ≥65**	0	+1

Comparison between derivation and validation settings

	**Derivation settings**	**Validation hospital cohorts**		
		**CliniCo**	**COVID-OLD**	**COVID-Predict**
**Country**	United Kingdom	The Netherlands	The Netherlands	The Netherlands
**Healthcare setting**	Hospital	Hospital	Hospital	Hospital
**Population**	• Adults (≥18 years)<br>• Confirmed COVID -19 diagnosis	• Older population (≥70 years)<br>• Confirmed COVID -19 diagnosis	• Older population (≥70 years)<br>• Confirmed COVID -19 diagnosis	• Older population (≥70 years)<br>• Confirmed COVID -19 diagnosis
**Predicted outcome and time of horizon**	In-hospital mortality	In-hospital mortality	In-hospital mortality	In-hospital mortality

Approximated in-hospital mortality risks

**CURB-65 score**	**Mortality score**
**0**	0.6%
**1**	2.7%
**2**	6.8%
**3**	14%
**4 to 5**	27.8%

Reference

W Lim, M.M van der Eerden, R Laing, W Boersma, N Karalus, G Town, S lewis, and J Macfarlane. Defining community acquired pneumonia severity on presentation to hospital: an international deviation and validation study. Thorax. 2003 May; 58(5):377-382. Doi: 10.1136/thorax.58.5.377.

8) SOFA score

Score calculation table

Physiological parameter	SOFA Score				
	0	1	2	3	4
**Partial pressure of oxygen / Fraction of inspired oxygen (mmHg)**	≥400	300-399	200-299	100-199 with respiratory support	<100 with respiratory support
**Platelets (10^3/ µL)**	≥150	100-149	50-99	20-49	<20
**Bilirubin µmol/L (mg/dL)**	<20 (1.2)	20-32 (1.2-1.9)	33-101 (2-5.9)	102-204 (6-11.9)	>204 (12)
**Mean arterial pressure mmHg**	≥70	<70	Low dose dopamine or any dose dobutamine	Low-medium dose noradrenalin or adrenalin; medium dose dopamine	High dose noradrenalin, or dopamine
**Glasgow Coma Scale score**	15	13-14	10-12	6-9	<6
**Creatinine, µmol/L(mg/dL)**	<110 (1.2)	110-170 (1.2-1.9)	171-299 (2.0-3.4)	300-440 (3.5-4.9)	>440 (5.0)
**Urine output, mL/day**	-	-	-	<500	<200

Comparison between derivation and validation settings

	**Derivation settings**	**Validation hospital cohorts**		
		**CliniCo**	**COVID-OLD**	**COVID-Predict**
**Country**	Unclear	The Netherlands	The Netherlands	The Netherlands
**Healthcare setting**	Hospital	Hospital	Hospital	Hospital
**Population**	• Adults (≥18 years)<br>• Confirmed COVID -19 diagnosis	• Older population (≥70 years)<br>• Confirmed COVID -19 diagnosis	• Older population (≥70 years)<br>• Confirmed COVID -19 diagnosis	• Older population (≥70 years)<br>• Confirmed COVID -19 diagnosis
**Predicted outcome and time of horizon**	ICU mortality	In-hospital mortality	In-hospital mortality	In-hospital mortality

Approximated in-hospital mortality risks

Mean SOFA score	Mortality risk
0 to 1.0	1.2%
1.1 to 2.0	5.4%
2.1 to 3.0	20%
3.1 to 4.0	36.1%
4.1 to 5.0	73.1%
> 5.1	84.4%

Reference

Vincent JL, Moreno R, Takala J, et al. The SOFA (Sepsis-related Organ Failure Assessment) score to describe organ dysfunction/failure. On behalf of the Working Group on Sepsis-Related Problems of the European Society of Intensive Care Medicine. In: Vol 22. 1996:707–710.
