# Supplementary material for: A study protocol of external validation of eight COVID-19 prognostic models for predicting mortality risk in older populations in a hospital, primary care, and nursing home setting
Source: Diagn Progn Res. 2023 Apr 4;7:8. doi: 10.1186/s41512-023-00144-2 (PMC10069944; doi:10.1186/s41512-023-00144-2)
Supplement: Supplementary file 3 — Additional file 3: Supplementary file 3. Predictor measurements in derivation and validation cohorts. [file 41512_2023_144_MOESM3_ESM.pdf]

Supplementary file 3: Predictor measurements in derivation and validation cohorts

Predictor measurements were extracted from electronic health records.

Supplementary Table 2: Predictor measurements in original derivation settings and validation cohorts

Predictors	Derivation settings		Validation settings
	**Unit and definition in derivation study**	**Timing of measurement in the derivation study**	**Definition and timing of measurement in primary care and nursing homes**
	**GAL-COVID-19 mortality**		
**Age**	Years	At admission	At admission
**Sex**	At birth		At admission
**Lymphoma/leukemia/Hodgkin's disease**	ICPC-2 codes B72, B73, B74	At PCR-confirmed covid-19 diagnosis	JHN/AHA/AHN and PHARMO: ICPC codes B72, B72.01, B72.02, B73, B74<br>Ysis: Information extracted from free text of patient history
**Liver disease**	ICPC-2 code D97	At PCR-confirmed covid-19 diagnosis	JHN/AHA/AHN and PHARMO: ICPC code D87<br>Ysis: Information extracted from free text of patient history
**Dementia**	ICPC-2 code P70	At PCR-confirmed covid-19 diagnosis	JHN/AHA/AHN and PHARMO: ICPC code P70<br>Ysis: Information extracted from free text of patient history
**Ischemic heart disease**	ICPC-2 codes K74, K75, K76	At PCR-confirmed covid-19 diagnosis	JHN/AHA/AHN and PHARMO: ICPC K74, K75, K76<br>Ysis: Information extracted from free text of patient history
**Chronic Obstructive Pulmonary Disease**	ICPC-2 code R95	At PCR-confirmed covid-19 diagnosis	JHN/AHA/AHN and PHARMO: ICPC R95<br>Ysis: Information extracted from free text of patient history
**Diabetes mellitus**	ICPC-2 codes T89, T90, W85	At PCR-confirmed covid-19 diagnosis	JHN/AHA/AHN and PHARMO: ICPC T90.01, T90.02, W84.02, W84.03<br>Ysis: Information extracted from free text of patient history
**Chronic kidney disease**	ICPC-2 code U99.01	At PCR-confirmed covid-19 diagnosis	JHN/AHA/AHN and PHARMO: ICPC U99.01<br>Ysis: Information extracted from free text of patient history

Predictors	Derivation settings		Validation settings
	**Unit and definition in derivation study**	**Timing of measurement in derivation study**	**Definition and timing of measurement in hospital cohorts**
	**GAL-COVID-19 mortality**		
**Age**	Years	At covid-19 diagnosis	Registered once at hospital admission

**Sex**	At birth		Registered once at hospital admission
**Lymphoma/leukemia/Hodgkin's disease**	ICPC-2 codes B72, B73, B74	At PCR-confirmed covid-19 diagnosis	CliniCo and COVID-PREDICT: Recorded in patient history under hematological malignancies
**Liver disease**	ICPC-2 code D97	At PCR-confirmed covid-19 diagnosis	CliniCo, COVID-PREDICT and COVID-OLD: Registered once at hospital admission
**Dementia**	ICPC-2 code P70	At PCR-confirmed covid-19 diagnosis	CliniCo, COVID-PREDICT and COVID-OLD: Registered once at hospital admission
**Ischemic heart disease**	ICPC-2 codes K74, K75, K76	At PCR-confirmed covid-19 diagnosis	CliniCo, COVID-PREDICT and COVID-OLD: Registered once at hospital admission
**Chronic Obstructive Pulmonary Disease**	ICPC-2 code R95	At PCR-confirmed covid-19 diagnosis	CliniCo, COVID-PREDICT and COVID-OLD: Registered once at hospital admission
**Diabetes mellitus**	ICPC-2 codes T89, T90, W85	At PCR-confirmed covid-19 diagnosis	CliniCo, COVID-PREDICT and COVID-OLD: Registered once at hospital admission
**Chronic kidney disease**	ICPC-2 code U99.01	At PCR-confirmed covid-19 diagnosis	CliniCo, COVID-PREDICT and COVID-OLD: Registered once at hospital admission
**4C-Mortality Score**			
**Age**	Years	At hospital admission	Registered once at hospital admission
**Sex**	Sex at birth		Registered once at hospital admission
**Chronic cardiac disease**	Not reported for original model	Day of admission to hospital	CliniCo, COVID-PREDICT and COVID-OLD: Registered once at hospital admission from electronic health record
**Chronic respiratory disease (excluding asthma)**	Not reported for original model	Day of admission to hospital	CliniCo, COVID-PREDICT and COVID-OLD: Registered once at hospital admission from electronic health record
**Chronic renal disease**	Not reported for original model	Day of admission to hospital	CliniCo, COVID-PREDICT and COVID-OLD: Registered once at hospital admission from electronic health record
**Mild to severe liver disease**	Not reported for original model	Day of admission to hospital	CliniCo, COVID-PREDICT and COVID-OLD: Registered once at hospital admission from electronic health record
**Dementia**	Not reported for original model	Day of admission to hospital	CliniCo, COVID-PREDICT and COVID-OLD: Registered once at hospital admission from electronic health record
**Chronic neurological conditions**	Not reported for original model	Day of admission to hospital	CliniCo, COVID-PREDICT and COVID-OLD: Registered once at hospital admission from electronic health record

**Connective tissue disease**	Not reported for original model	Day of admission to hospital	CliniCo, COVID-PREDICT and COVID-OLD: Registered once at hospital admission from electronic health record
**Diabetes mellitus**	Not reported for original model	Day of admission to hospital	CliniCo, COVID-PREDICT and COVID-OLD: Registered once at hospital admission from electronic health record
**HIV or AIDS**	Not reported for original model	Day of admission to hospital	CliniCo, COVID-PREDICT and COVID-OLD: Registered once at hospital admission from electronic health record
**Malignancy**	Not reported for original model	Day of admission to hospital	CliniCo, COVID-PREDICT and COVID-OLD: Registered once at hospital admission from electronic health record
**Obesity**	Clinician defined (not further defined)	Day of admission to hospital	COVID-OLD and COVID-PREDICT: History recorded at hospital admission<br>CliniCo: Computed based on BMI using weight and height
**Respiratory rate**	Breaths per minute	Day of admission to hospital	CliniCo and COVID-OLD: Registered as monitoring data<br>COVID-OLD: recorded in Modified Early Warning Score (MEWS) at admission
**Peripheral oxygen saturation on room air**	%	Day of admission to hospital	CliniCo and COVID-PREDICT: Measured at hospital admission<br>COVID-OLD: recorded in Modified Early Warning Score (MEWS) at admission
**Glasgow Coma Scale**	Score 15 or <15	Day of admission to hospital	CliniCo: Recorded by health professional at hospital admission ACVPU score<br>COVID-OLD: Recorded by health professional regularly in intensive care and hospital monitored patients
**Urea**	Mmol/L	Day of admission to hospital	Measured at hospital admission
**C-reactive protein**	Mg/dL	Day of admission to hospital	Measured at hospital admission
		**Xie model**	
**Age**	Years	Not Specified	Registered once at hospital admission
**Lactate dehydrogenase**	IU/L	Not Specified	Measured at hospital admission
**lymphocyte count**	10^9 cells/L	Not Specified	Measured at admission
**Oxygen saturation**	%	Not Specified	Measured at admission<br>COVID-OLD: recorded in Modified Early Warning Score (MEWS) at admission
		**Wang model**	
**Age**	Years	At hospital admission	Registered once at hospital admission
**History of hypertension**	Recorded at hospital admission	At hospital admission	Registered once at hospital admission
**History of chronic heart disease**	Recorded at hospital admission	At hospital admission	Registered once at hospital admission
		**NEWS2+ model**	

**Respiratory rate**	Breaths/minute	First available measure up to 48 h following hospital admission	Registered once at hospital admission<br>COVID-OLD: recorded in Modified Early Warning Score (MEWS) at admission
**Oxygen saturation**	%	First available measure up to 48 h following hospital admission	Measured at hospital admission<br>COVID-OLD: recorded in Modified Early Warning Score (MEWS) at admission
**Heart rate**	Beats/minute	First available measure up to 48 h following hospital admission	Registered once at hospital admission<br>COVID-OLD: recorded in Modified Early Warning Score (MEWS) at admission
**Systolic blood pressure**	mmHg	First available measure up to 48 h following hospital admission	Part of monitoring data at hospital admission
**Body temperature**	Degree Celsius	First available measure up to 48 h following hospital admission	Part of monitoring data at hospital admission<br>COVID-OLD: recorded in Modified Early Warning Score (MEWS) at admission
**Alertness**	AVPU score	First available measure up to 48 h following hospital admission	Recorded by health professional at hospital admission ACVPU score<br>COVID-OLD: Recorded by health professional regularly in intensive care and hospital monitored patients
**Supplemental oxygen flow rate**	L/min	First available measure up to 48 h following hospital admission	Reported by health professional at hospital admission<br>COVID-OLD: recorded in Modified Early Warning Score (MEWS) at admission
**Urea**	Mmol/L	First available measure up to 48 h following hospital admission	Measured at hospital admission
**Age**	Years	First available measure up to 48 h following hospital admission	Registered once at hospital admission
**C-reactive protein**	Mg/L	First available measure up to 48 h following hospital admission	Measured at hospital admission and repeated 3-4 times a week if indicated
**Estimated glomerular filtration rate**	ml/min	First available measure up to 48 h following hospital admission	Computed using MDRD equation using age, sex, and creatinine values
**Neutrophil count**	10^9 cells/L	First available measure up to 48 h following hospital admission	Measured at hospital admission and repeated 3-4 times a week if indicated
**Neutrophil/lymphocyte ratio**		First available measure up to 48 h following hospital admission	Computed using serum Neutrophil and lymphocytes levels
	**SOFA score**		
**Arterial oxygen tension**	MmHg	24-hour period after the onset of infection/admission for infection	Calculated using arterial or venous blood gas test levels at hospital admission
**Fraction of inspired oxygen ratio**	%<br>24-hour period after the onset of	24-hour period after the onset of	Calculated using arterial oxygen partial pressure and fractional

	infection/admission for infection	infection/admission for infection	inspired oxygen measured at hospital admission
**Glasgow Coma Scale**	Score 15 or <15	24-hour period after the onset of infection/admission for infection	Recorded by health professional at hospital admission
**Mean arterial pressure**	MmHg	24-hour period after the onset of infection/admission for infection	Computed using systolic and diastolic blood pressure measured at hospital admission
**Administration of vasopressors**	Dopamine ≤5 or Dobutamine (any dose) / Dopamine >5, Epinephrine ≤0.1, or norepinephrine ≤0.1 / Dopamine >15, Epinephrine >0.1, or norepinephrine >0.1	24-hour period after the onset of infection/admission for infection	CliniCo and COVID-OLD: Patient information at admission checked for vasopressors
**Serum creatinine**	mg/dL	24-hour period after the onset of infection/admission for infection	Measured at hospital admission
**Bilirubin**	mg/dL	24-hour period after the onset of infection/admission for infection	Measured at hospital admission
**Platelet count**	10^9 cells/L	24-hour period after the onset of infection/admission for infection	Measured at hospital admission
		**APACHE-II Score**	
**Body temperature**	Rectal temperature degree Celsius	The worst parameters in the first 24 hours of hospitalization	Part of monitoring data at hospital admission<br>COVID-OLD: recorded in Modified Early Warning Score (MEWS) at admission
**Heart rate**	Beats/minute	The worst parameters in the first 24 hours of hospitalization	Part of monitoring data at hospital admission<br>COVID-OLD: recorded in Modified Early Warning Score (MEWS) at admission
**Breathing rate**	Breaths/minute	The worst parameters in the first 24 hours of hospitalization	Part of monitoring data at hospital admission
**Mean arterial pressure**	MmHg	The worst parameters in the first 24 hours of hospitalization	Computed using systolic and diastolic arterial pressure
**Arterial oxygen tension**	MmHg	The worst parameters in the first 24 hours of hospitalization	Calculated using arterial or venous blood gas test levels at hospital admission
**pH**		The worst parameters in the first 24 hours of hospitalization	Measured at hospital admission
**Potassium**	Mmol/L	The worst parameters in the first 24 hours of hospitalization	Measured at hospital admission
**Sodium**	Mmol/L	The worst parameters in the first 24 hours of hospitalization	Measured at hospital admission
**Creatinine**	Micromol/L	The worst parameters in the first 24 hours of hospitalization	Measured at hospital admission

**Haematocrit**	%	The worst parameters in the first 24 hours of hospitalization	Measured at hospital admission<br>COVID-PREDICT: Not measured
**Leucocyte count**	10^9 cells/L	The worst parameters in the first 24 hours of hospitalization	Measured at hospital admission
**Glasgow Coma Scale**	Score 15 or <15	The worst parameters in the first 24 hours of hospitalization	COVID-OLD: Recorded by health professional regularly in intensive care and hospital monitored patients
**Age**	Years	The worst parameters in the first 24 hours of hospitalization	Registered once at hospital admission
**Chronic health evaluation**	Binary: history of surgery, history of organ insufficiency, immunocompromised state	24-hour period after the onset of infection/admission for infection	Recorded once by health professional at hospital admission<br>COVID-PREDICT: Not measured
	**CURB-65 Score**		
**Alertness**	AVPU score<br>AVPU, Alert vs. Confused/not alert (ACVPU classification, where A is alert, and CVPU are grouped as one score)	Not reported for original model	Recorded by health professional at hospital admission or AVPU score in cohorts
**Urea**	Mmol/L	Not reported for original model	Measured at hospital admission
**Respiratory rate**	Breaths/minute	Not reported for original model	Part of monitoring data at hospital admission
**Systolic blood pressure**	MmHg	Not reported for original model	Part of monitoring data at hospital admission
**Diastolic blood pressure**	MmHg	Not reported for original model	Part of monitoring data at hospital admission
**Age**	Years	Not reported for original model	Registered once at hospital admission
