# Supplementary material for: A study protocol of external validation of eight COVID-19 prognostic models for predicting mortality risk in older populations in a hospital, primary care, and nursing home setting
Source: Diagn Progn Res. 2023 Apr 4;7:8. doi: 10.1186/s41512-023-00144-2 (PMC10069944; doi:10.1186/s41512-023-00144-2)
Supplement: Supplementary file 4 — Additional file 4: Supplementary file 4. TRIPOD checklist for validation of prediction model [file 41512_2023_144_MOESM4_ESM.pdf]

Supplementary file 4: TRIPOD checklist for validation of prediction model

Supplementary table 3: TRIPOD checklist for the current external validation study of COVID-19 prognostic models in different healthcare settings

Section/Topic		Checklist Item	Page
**Title and abstract**			
**Title**	1	Identify the study as developing and/or validating a multivariable prediction model, the target population, and the outcome to be predicted.	1
**Abstract**	2	Provide a summary of objectives, study design, setting, participants, sample size, predictors, outcome, statistical analysis, results, and conclusions.	2
		**Introduction**	
**Background and objectives**	3a	Explain the medical context (including whether diagnostic or prognostic) and rationale for developing or validating the multivariable prediction model, including references to existing models.	3
	3b	Specify the objectives, including whether the study describes the development or validation of the model or both.	3
		**Methods**	
**Source of data**	4a	Describe the study design or source of data (e.g., randomized trial, cohort, or registry data), separately for the development and validation data sets, if applicable.	4
	4b	Specify the key study dates, including start of accrual; end of accrual; and, if applicable, end of follow-up.	4
**Participants**	5a	Specify key elements of the study setting (e.g., primary care, secondary care, general population) including number and location of centres.	4<br>Table 2
	5b	Describe eligibility criteria for participants.	4
	5c	Give details of treatments received, if relevant.	NA
**Outcome**	6a	Clearly define the outcome that is predicted by the prediction model, including how and when assessed.	5
	6b	Report any actions to blind assessment of the outcome to be predicted.	5
**Predictors**	7a	Clearly define all predictors used in developing or validating the multivariable prediction model, including how and when they were measured.	5
	7b	Report any actions to blind assessment of predictors for the outcome and other predictors.	NA
**Sample size**	8	Explain how the study size was arrived at.	7
**Missing data**	9	Describe how missing data were handled (e.g., complete-case analysis, single imputation, multiple imputation) with details of any imputation method.	6
**Statistical analysis methods**	10c	For validation, describe how the predictions were calculated.	5<br>Supple ment 3
	10d	Specify all measures used to assess model performance and, if relevant, to compare multiple models.	6
	10e	Describe any model updating (e.g., recalibration) arising from the validation, if done.	7
**Risk groups**	11	Provide details on how risk groups were created, if done.	NA
**Development vs. validation**	12	For validation, identify any differences from the development data in setting, eligibility criteria, outcome, and predictors.	6<br>supple ment 3

		**Results**	
**Participants**	13a	Describe the flow of participants through the study, including the number of participants with and without the outcome and, if applicable, a summary of the follow-up time. A diagram may be helpful.	NA
	13b	Describe the characteristics of the participants (basic demographics, clinical features, available predictors), including the number of participants with missing data for predictors and outcome.	NA
	13c	For validation, show a comparison with the development data of the distribution of important variables (demographics, predictors and outcome).	NA
**Model performance**	16	Report performance measures (with CIs) for the prediction model.	NA
**Model-updating**	17	If done, report the results from any model updating (i.e., model specification, model performance).	NA
		**Discussion**	
**Limitations**	18	Discuss any limitations of the study (such as nonrepresentative sample, few events per predictor, missing data).	8
**Interpretation**	19a	For validation, discuss the results with reference to performance in the development data, and any other validation data.	8
	19b	Give an overall interpretation of the results, considering objectives, limitations, results from similar studies, and other relevant evidence.	8
**Implications**	20	Discuss the potential clinical use of the model and implications for future research.	9
		**Other information**	
**Supplementary information**	21	Provide information about the availability of supplementary resources, such as study protocol, Web calculator, and data sets.	17-38
**Funding**	22	Give the source of funding and the role of the funders for the present study.	9
